# A mouse pancreatic organoid model to compare PD-L1 blocking antibodies

**DOI:** 10.1080/19420862.2022.2139886

**Published:** 2022-11-05

**Authors:** Guangyuan Li, Susmita Ghosh, JuMe Park, Hyunsu Shin, Mamatha Garige, Gregory Reaman, Carole Sourbier

**Affiliations:** a Division of Biotechnology Review and Research 1, Office of Biotechnology Products, Office of Pharmaceutical Quality, Center for Drug Evaluation and Research, US Food and Drug Administration, Silver Spring, Maryland, USA; b Oncology Center of Excellence, US Food and Drug Administration, Silver Spring, Maryland, USA

**Keywords:** Immune checkpoint inhibitors, PD-L1, diabetes, metabolism, pancreatic organoids

## Abstract

Immune checkpoint inhibitors (ICIs) have changed the therapeutic landscape for cancer patients, but diabetes, a rare, severe immune-related endocrinopathy, is linked to ICI therapy. It is unclear whether glycosylation of ICIs may play a role in the development of this adverse event and how the physiological effects of different ICIs on pancreatic cells should be evaluated. We used a mouse pancreatic organoid model to compare three PD-L1 blocking antibodies in the presence or absence of IFNγ using a metabolic bioanalyzer. Modulation of ICI glycosylation altered its metabolic effects on mouse pancreatic organoids, suggesting that this model could be used to monitor and compare ICIs and to study the mechanisms underlying the development of IC-mediated diabetes.

## Introduction

Immune checkpoint inhibitors (ICIs) have changed the therapeutic landscape for numerous individuals with cancer.^1^ They are also responsible for significant severe adverse events, such as immune-related endocrinopathies.^2–4^ The manifestations and natural history of these endocrinopathies are unusual, as they are often irreversible and will require lifelong management. It remains unclear what leads to the loss of insulin-producing cells and whether these adverse events are due to the intrinsic biological effect of the therapy (i.e., target dependent) or whether they could be due to a product quality attribute, such as the glycosylation of a therapeutic monoclonal antibody (mAb). Immune checkpoints are key in maintaining control of the immune system.^5^ They include PD-1 and PD-L1, CTLA4 and LAG-3, and have been therapeutic targets for numerous oncology indications to enhance the immune response against tumor cells. These checkpoints also have cellular intrinsic effects.^6–8^ For example, PD-L1 expression mediates some of the interferon γ (IFNγ)’s effects in a ligand-independent manner and regulates the cellular metabolism of kidney tumor cells *in vitro*.^8^ IFNγ is a cytokine that has been shown to have both pro- and anti-tumorigenic effects depending on the cell type and the microenvironment. It has been shown to play a key role in the induction of signaling pathways related to inflammation and in the expression of immune checkpoints such as PD-L1.^9,10^ IFNγ is considered a potential marker for ICI therapeutic response in some settings,^11^ and may play some roles in the development of ICI-mediated adverse events, as suggested by a recent small-cohort study that shows that CD8+ tissue resident memory T cells, which produce IFNγ, are involved in the development of ICIs induced colitis.^12^ In addition, IFNγ plays an important role in inflammation and autoimmune disease.^13^ For example, it has been shown to induce PD-L1 expression on islet β cells to limit their destruction by autoreactive T cells and to be associated with chronic pancreatitis.^14,15^

Organoids have been used in bioassays, drug development, and nonclinical studies for several years. They have the advantage of recapitulating key features of an organ within a controlled environment, providing an intermediate model between classical 2-dimensional cell culture and *in vivo* studies.^16–20^ They can also be used as tools to understand disease initiation and progression and to understand the mechanisms underlying the mechanism of action of therapeutics or the development of adverse events.

In this study, we assessed whether a mouse pancreatic organoid model could be used to compare PD-L1 inhibitors, which could provide clues as to whether quality attributes of ICIs may contribute to the development of immune-related diabetes.

## Results

**Figure 1.**
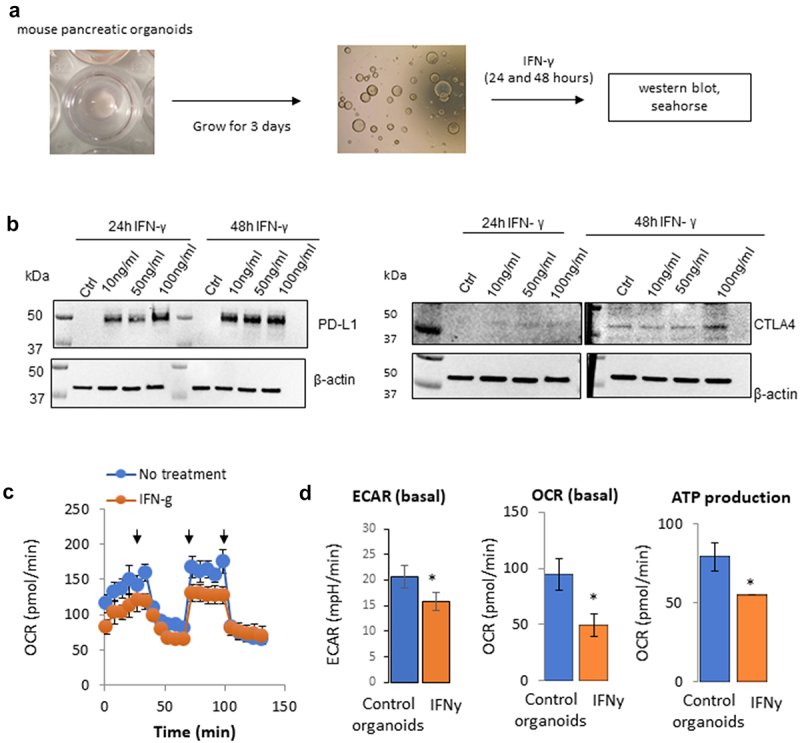
Effect of IFNγ on the metabolism of mouse pancreatic organoids. a) Schematic representation of the timeline using mouse pancreatic organoids. b) Immunoblotting of PD-L1 and CTLA4 protein expression following a concentration range of IFNγ exposure at 24 hr and 48 hr. c) Metabolic profiling of mouse pancreatic organoids following IFNγ exposure (24 hr, 10 ng/mL). Arrows indicate respectively injection of oligomycin, FCCP, rotenone/antimycin. d) Extracellular acidification rate (ECAR), basal oxygen consumption rate (OCR), and ATP production were measured using a metabolic bioanalyzer following IFNγ exposure (24 hr, 10 ng/mL). *p < .05.

IFNγ and PD-L1 signaling pathways and interactions have been studied in immune and tumor cells, but less is known about their role in other settings. Because of the reported role of IFNγ on pancreatic cells and its connection with the PD-L1 signaling,^9,10,14,15^ we assessed whether, similarly to tumor cells, pancreatic organoids (derived from the pancreas of C57BL/6 mice) expressed PD-L1 following IFNγ exposure and whether it leads to a metabolic rewiring toward aerobic glycolysis.^8^ Preparation of mouse pancreatic organoids was performed as depicted in Figure 1a and described in the methods. Briefly, the cells were plated in 6-well plates in Matrigel. After 3 days, organoids were observed and cultured for an additional 24 or 48 hours in the presence or absence of IFNγ at a concentration range indicated in Figure 1b. Proteins were then extracted, and expression of PD-L1 and CTLA4 was assessed by immunoblotting (Figure 1b). The protein expression levels of PD-L1 and CTLA4 were not detectable in the mouse pancreatic organoids at baseline control. However, PD-L1 expression was strongly induced after 24 and 48 hours at all IFNγ concentrations tested (10, 50, and 100 ng/mL). The expression of CTLA4 was minimally noticeable at 24 hours and was induced after 48 hours of IFNγ exposure at 100 ng/mL. Since PD-L1 expression was easily inducible in this mouse pancreatic organoid model and PD-L1 is known to regulate cellular metabolism, we compared the metabolic profile of mouse pancreatic organoids in the presence or absence of IFNγ. As shown in Figure 1c-d, both the oxygen respiration rate (OCR) and the extracellular acidification rate (ECAR) were decreased following IFNγ exposure, which correlated with a decrease in ATP production. IFNγ decreased the overall metabolism of these organoids, which could be an early sign of cell apoptosis or senescence. While it differs from observations from us and others in tumor cells, these data are consistent with other reports showing that IFNγ could induce apoptosis in certain settings.^21^

**Figure 2.**
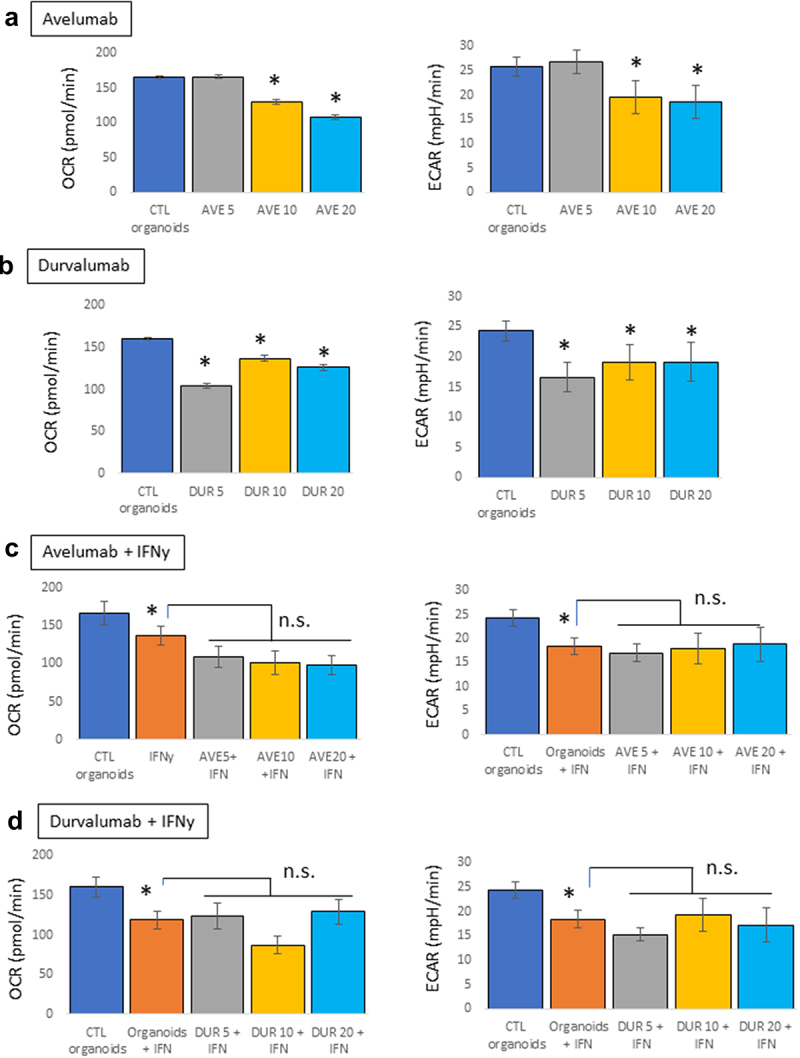
Metabolic effects of PD-L1 blocking antibodies on mouse pancreatic organoids. Oxygen consumption rates (OCR) and extracellular acidification rates (ECAR) were measured using a seahorse bioanalyzer following treatment of the organoids with avelumab or durvalumab (5, 10, or 20 μg/mL, 24 hr) with and without IFNγ (10 ng/mL). a) Effects of avelumab (AVE) without IFNγ. b) Effect of durvalumab (DUR) without IFNγ. c) Effects of avelumab (AVE) with IFNγ. d) Effect of durvalumab (DUR) with IFNγ. *p < .05; n.s.: not significant. Experiments were repeated three times with 4–5 technical replicates per plates.

Immune-related diabetes following ICIs therapy is due to the death of insulin-producing β cells. This mouse pancreatic organoid model, by monitoring concomitantly the energetics of the organoids and their health, may provide valuable information as to which external factors or therapy might induce endocrine-related adverse events. Therapeutic mAbs are complex structures with biological activity that may rely on Fc-effector function linked to their glycosylation status, or the IgG type used as the backbone of the antibody.^22–24^ To differentiate between the effects linked to the binding to PD-L1 and potential effector functions, we tested the metabolic effects of two anti-PD-L1 antibodies, avelumab, and durvalumab, in the presence or absence of IFNγ (10 ng/mL). Organoids were treated with a concentration range of the PD-L1 blocking antibodies (5, 10, and 20 µg/mL, 24 hours). No effects were observed with avelumab at 5 µg/mL, but the concentrations of 10 and 20 µg/mL decreased both OCR and ECAR of the organoids (Figure 2a). Durvalumab induced a decrease in OCR and ECAR compared to control organoids at all three concentrations (Figure 2b). In the presence of IFNγ, none of the mAbs induced any additional effects relative to those induced by IFNγ (Figure 2c). Because these mAbs are PD-L1-blocking antibodies, our data suggest that the metabolic effects of these mAbs on mouse pancreatic organoids in the absence of IFNγ are independent of their binding to PD-L1 and might be mediated by other characteristics. Both avelumab and durvalumab are glycosylated; avelumab has been shown to have intact Fc function and to induce antibody-dependent cell-mediated cytotoxicity (ADCC),^25^ while durvalumab has a triple-mutated (L234F, L235E, P331S) Fc region with reduced Fc functions.^26^ Therefore, in the absence of PD-L1 expression (and absence of IFNγ), the metabolic effects observed on the pancreatic organoids might be linked to the glycosylation of the mAbs. However, in the presence on IFNγ, it is unclear if the mAbs have any effect or if their effects might be hidden by the overall decrease in metabolism of the organoids driven by IFNγ.

**Figure 3.**
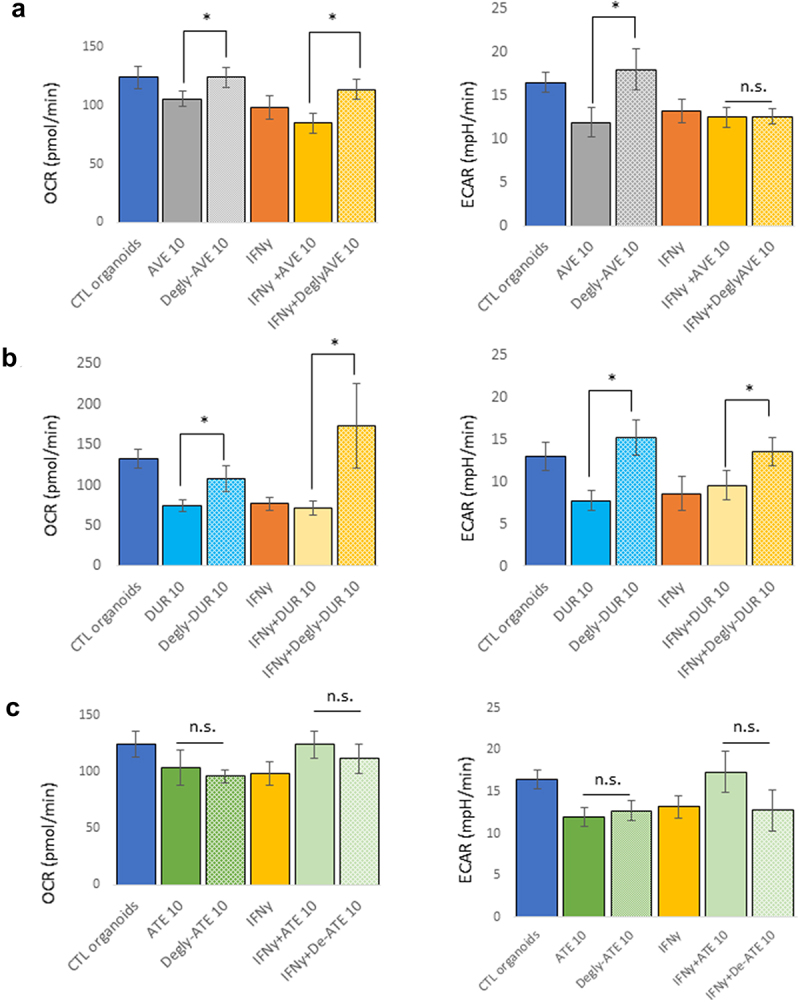
Effect of deglycosylation of mAbs on the metabolism of mouse pancreatic organoids. Avelumab (a) and Durvalumab (b) were deglycosylated, and cells were treated with glycosylated or deglycosylated mAbs (10 μg/mL, 24 hr) and with or without IFNγ (10 ng/mL). c) Atezolizumab is not glycosylated and was used as a control. “Degly-ATE” indicates that atezolizumab was subjected to the deglycosylation protocol as a control. *p < .05; n.s.: not significant. Experiments were repeated three times with 3–4 technical replicates per plates.

To further assess whether this metabolic analysis of mouse pancreatic organoids could be reflective of the physiological effect of the glycosylation status of these mAbs, we repeated these experiments after deglycosylation of avelumab and durvalumab, and included atezolizumab, an IgG1 mAb that lacks glycosylation due to a mutation (N297A) in its Fc,^23^ as control. Although aglycosylated, atezolizumab was subjected to the deglycosylation protocol, which is indicated in Figure 3 as “Degly-Ate”, to ensure that this protocol did not affect the biological integrity of the mAb. Organoids were then treated with the glycosylated and deglycosylated mAbs at 10 µg/mL for 24 hours, in the presence or absence of IFNγ (10 ng/mL). In the absence of IFNγ, the metabolic effects of avelumab and durvalumab on the OCR and ECAR of the organoids were reversed by their deglycosylation (Figure 3a) and b. In the presence of IFNγ, the effect of deglycosylated mAbs were different: although both deglycosylated avelumab and durvalumab increased the organoids OCR, deglycosylated avelumab had no effects on ECAR, while deglycosylated durvalumab reversed the organoids ECAR to levels displayed by organoids without IFNγ exposure. Taken together, these data further support that glycosylation of avelumab and durvalumab modulates the metabolism and health of pancreatic organoids, potentially independently of Fc-mediated antibody effector functions. Because the metabolic effect of IFNγ was partially reversed using deglycosylated mAbs, it is possible that IFNγ and a functional effect of glycosylation of the mAbs might share common intracellular signaling pathways. These data also indicate that the readout of this bioassay is independent of PD-L1 expression and reflects an effect of glycosylation of the mAbs on the metabolism of mouse pancreatic organoids.

## Discussion

In this report, we assessed the effects of ICIs on mouse pancreatic organoids using the organoids metabolism as a readout of viability. By comparing three IgG1 PD-L1 blocking antibodies, we found that the metabolic changes observed were independent of PD-L1 binding, as they could be observed in the absence of detectable PD-L1 expression. Deglycosylation of the mAbs reversed at least partially the observed effects, suggesting that differences in glycosylation status between mAbs beyond PD-L1 blocking mAbs could potentially be detected using that model.

Glycosylation and post-transcriptional modifications of mAbs are complex and could lead to diverse and tissue-specific effects.^24,27,28^ Fcγ receptors play a role in the regulation of immunity and inflammation, and have been linked to the development of diabetes in a NOD mouse model.^29^ It is unclear in our model whether the metabolic effects observed with avelumab may be mediated by Fcγ receptors or how glycosylation of the ICIs is affecting the health of the organoids. Monitoring the energetic profile of organoids using a metabolic bioanalyzer provides an opportunity to study complex mechanisms and to compare complex therapeutic agents. Although more studies are needed to link the metabolic effect observed with clinical adverse events and to fully characterize the organoids, monitoring the metabolism and viability of mouse pancreatic organoids using a metabolic bioanalyzer provides a platform to further study the effect of antibody post-transcriptional modifications on endocrine-related adverse events and to compare their biological effects. In addition, because the effects observed were PD-L1 independent, it would be interesting to see whether other glycosylated mAbs, such as CTLA4 or PD-1 blocking antibodies, may have an effect on the metabolism of mouse pancreatic organoids. Use of this model could lead to a better understanding of molecular mechanisms that drive the development of adverse events by defining the potential role of structural attributes of mAbs and could improve the design, control, and safety of antibody therapeutics.

## Materials and Methods

### Antibodies

PD-L1-blocking mAbs avelumab (EMD Serono/Pfizer), durvalumab (MedImmune/AstraZeneca), and atezolizumab (Genentech/Roche) were purchased via McKesson Specialty Health (Scottsdale, Arizona).

### Culture of mouse pancreatic organoids

Mouse pancreatic organoids were purchased from StemCell Technologies (#70933, Cambridge, MA) and cultured in 6-well plates using PancreaCult Organoid Growth Medium (Mouse, #06040, StemCell Technologies) following the manufacturer’s instructions. The expansion and cryopreservation protocols are detailed in Clinton and McWilliams-Koeppen.^30^ Organoid growth and viability were controlled at each passage or collection by cell counting and trypan blue staining following the dissociation of organoids contained in representative wells (1–2 wells for simple passage, 2–3 wells for experiments or storage). Organoids were passaged up to 10 times.

### Immunoblots

Matrigel domes were broken by mixing them 20 times in 1 mL cold phosphate-buffered saline (PBS) using a P1000 pipette. Content was then transferred into a 15 mL conical tube on ice. Wells were washed with cold PBS (about 1 mL) to capture any remaining organoid pieces. Tubes were centrifugated at 290 g for 5 min at 3–8°C. Once the Matrigel has been eliminated, the supernatant was discarded and RIPA lysis buffer containing protease inhibitor cocktail (#4693116001, Roche) was added. Whole-cell lysates were quantified using a BCA protein assay kit (#23227, ThermoScientific). Twenty micrograms of total protein were used. The blots were probed with primary antibodies against PD-L1 (#ab233482, Abcam) or CTLA4 (#ab237712, Abcam), and beta-actin (#4970, Cell Signaling). ECL-Plus kit was used as a chemiluminescence substrate (#32132, ThermoScientific), and detection was performed using the ChemiDoc MP system (Bio-Rad).

### Measure of oxygen consumption and extracellular acidification rates using a Seahorse Bioanalyzer

Metabolism of mouse pancreatic organoids was assessed using the Seahorse XF96 Analyzer (Agilent Seahorse Bioscience, CA). A Seahorse XF Sensor Cartridge was hydrated 24 hours prior to the assay in a non-CO_2_ 37°C incubator. On the day of the experiment, after removal of the Matrigel, organoids were washed 3 times and 50 organoids were plated in 96-well XF Plate with 200 μL of unbuffered XF Assay Media supplemented with 10 mM glucose, 2 mM sodium pyruvate, and 2 mM glutamine (adjusted to pH 7.4) and incubated in non-CO2 37°C incubator for 1 hour prior to performing the analysis in the analyzer.

### Deglycosylation of therapeutic antibodies

Deglycosylation of avelumab and durvalumab was performed according to the manufacturer’s protocol (Promega, #V483A). Atezolizumab, which is not glycosylated^23^ and was used as a control. Protein deglycosylation was performed using non-denaturing conditions for mass spectrometry with PNGase F. Briefly, 20 µg of mAbs was diluted in 50 mM ammonium bicarbonate (pH 7.8) to a final volume of 18 µL, then two µL of recombinant PNGase F was added and incubated at 37°C for 18 hours.

### Statistical methods

The Student's t-test was performed. p < 0.05 was considered significant.

## Abbreviations

ADCC, Antibody-dependent cell-mediated cytotoxicity; CTLA 4, Cytotoxic T-lymphocyte-associated protein 4; ICIs, Immune checkpoint inhibitors; IFNγ, interferon γ; mAbs, monoclonal antibodies; NOD, Non-obese diabetic; PD-L1, Programmed death-ligand 1; PNGase F, Peptide:N-glycosidase F.
